# Uphill Shifts of Fungal Fruiting Due to Climate Change at the Polar Urals

**DOI:** 10.3390/microorganisms9091892

**Published:** 2021-09-06

**Authors:** Anton G. Shiryaev

**Affiliations:** Vegetation and Mycobiota Biodiversity Department, Institute of Plant and Animal Ecology UB RAS, 8 March str. 202, 620144 Ekaterinburg, Russia; anton.g.shiryaev@gmail.com

**Keywords:** biodiversity, fungal ecology, cryophilous organisms, dynamics, climate change, permafrost, biogeography, vegetation, treeline, decomposition, Arctic

## Abstract

Due to the ongoing climatic changes in the Arctic, the ranges of many plants and animal species are rising higher into the mountains, into the treeline; however, such studies are rare for fungi. The 60-year fruiting dynamics of 66 species of Agaricomycetous macrofungi has been studied along the altitudinal transect located on the slope of Slantsevaya Mountain (Polar Urals, Russia). It has been found that the three basic trophic groups (mycorrhizal, saprobes on litter and soil, and saprobes on wood) fruit higher in the mountains. Additionally, for most of the studied species, a tendency towards upward displacement of fruiting was revealed. The rise in fruiting for saprobes on litter and soil was the most obvious. Mycorrhizal fungi associated with woody plants showed the least uplifting effect. Fungal species that were characterized by fruiting higher up the mountainside half a century ago show stronger upward shifts compared to species previously bearing fruit only at the mountain foot. Probably, such a reaction of the aboveground mycobiota is similar to the processes occurring in the soil, which are associated with an active increase in the decomposition rate of the litter, an increase in the depth of permafrost thawing, and a significant redistribution of the soil water balance. On the other hand, the rise of fungi is associated with an increase of plant biomass in the middle and upper parts, which are the most important sources of fungal nutrition.

## 1. Introduction

In terrestrial ecosystems, fungi are the main agents in the processes of decomposition and nutrient cycling as well as the consumption of nutrients by plants [1]. Hence, they have a vital impact on ecosystem processes and the carbon cycle on Earth. Changes in the productivity and phenology of fungal fruit bodies may provide clues to changes in fungal activity, but understanding these changes in light of climate change, especially in high latitudes, is an unresolved challenge for ecologists.

The climate in the Arctic is warming two to three times faster than the average rate on the planet [2,3]. Various groups of mycobiota actively respond to such climatic processes: species of forest mycobiota expand their range to the north and climb the mountains, but Arcto-Alpine species reduce their range [4]. In the subarctic and subalpine regions, the number of forest mycorrhizal species is increasing, while the number of cryophilic saprobes on the litter is decreasing [5,6]. The degradation of permafrost leads to the northward movement of “southern” woody plants, which are accompanied in the Subarctic by many forest wood-destroying fungal species that have not previously been registered beyond the Arctic Circle [7].

A growing number of articles are using genetic methods to study the response of mycelium and soil spores to climate change in the Arctic, i.e., “potential” diversity [4,5,6,7,8,9]. Nevertheless, the data describing how the “potential” diversity correlates with the “actual” one (fruit bodies of Agaricomycetous macromycetes) are still scarce. 

With regards to the identification of the macromycetes species composition in the Russian Arctic, the Polar Urals mountains located on the border of Europe and Asia have been studied best [10,11,12,13,14,15]. Over the past century, in the Polar Urals, the average annual air temperature increased by 2 °C and the average annual precipitation increased by 88 mm, as a result of which the growing season has increased by 7 days. As a result of the above bioclimatic parameters, the upper border of the forest has risen by 40–60 m [15,16] (Figures S1 and S2: Table S1), and the biomass of woody plants has increased tenfold [15,17]. New, typically forest species of mosses and vascular plants have appeared [18,19,20,21]; borealization of the fauna is taking place [22].

The question of the study is whether there has been an upward fruiting of macromycetes on Slantsevaya Mountain (the Polar Urals) over the last 60 years. What trophic groups of macromycetes (mycorrhizal, saprobes on soil and litter, saprobes on wood) have climbed the mountain most of all? Is the greatest uplift characteristic of the species that have previously fruited mainly in the upper and middle part of the slope or those fruiting at the mountain foot?

## 2. Material and Methods

### 2.1. Area of Investigation

**Figure 1:**
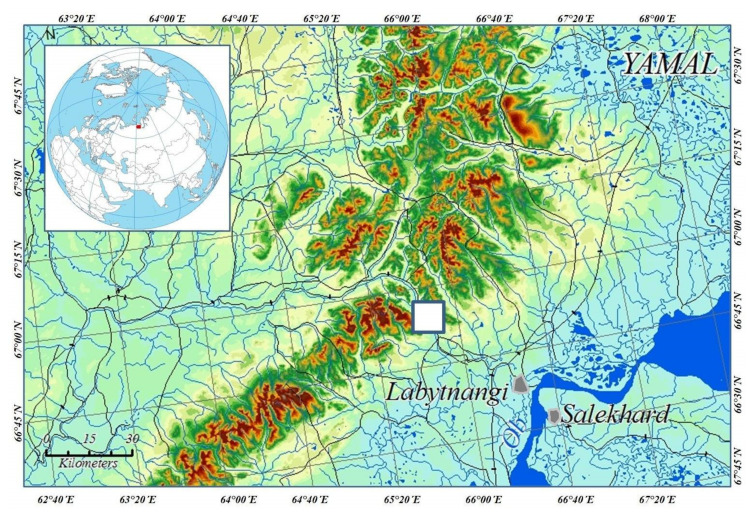
Map of the investigated area in the Polar Urals. The study area is marked as a white square.

The studies were carried out on the southwestern slope of Slantsevaya Mountain (N 66°54′; E 65°44′), located on the eastern macroslope of the Polar Urals [16], in the Yamal-Nenetsk Autonomous District (Figure 1). At its foot, in the valley of the Sob River (70 to 90 m a. s. l.), there are forest thickets representing northern boreal vegetation with a predominance of *Larix sibirica* Lebed., *Picea obovata* Lebed. with a large percentage of *Salix* spp., and *Betula pendula* Roth. The mountain slope is also covered by the spruce–larch dominated north-boreal forest, and the timberline is represented by forest-tundra vegetation (*L. sibirica*, *P. obovata*, *Duschekia fruticosa* (Rupr.) Pouzar, *Sorbus sibirica* Hedl., *Betula nana* L.). From the foot to the timberline, the mountain’s altitude ranges from 100 to 310 m above sea level. Higher up, there is mountain tundra (320-410 m above sea level) with varied moss and lichen cover, mountain bogs and thickets of *B. nana*, *Salix lanata* L., and *S. glauca* L. shrubs. In other words, the altitudes range on the transect from 70 to 410 m above sea level. The transect is 2.5 km long and 0.5 km wide. Consequently, the study area covers an area of 1.25 km^2^.

There is no economic activity on the mountain slopes. In 1940–1950, in the valley of the Sob River, there was a small village of builders of the Vorkuta-Labytnangi railway crossing the Polar Urals from west to east. Two people permanently live near the railway now.

### 2.2. Fungi Sampling

Macromycetes have been collected on Slantsevaya Mountain since 1960, where the staff of the Institute of Plant and Animal Ecology (Ural Branch of the Russian Academy of Sciences) laid long-term model plots for recording the species richness and macromycetes abundance [10]. Until now, there have been metal signs on the trees with the designation of the plot numbers, which helped us to identify the altitude level at which the studies were carried out in the 1960–1970s. There is an extensive list of publications [10,11,12,13,14,15,16], as well as a large amount of collection material. In 1996, participants in the Fifth International Symposium on Arcto-Alpine mycology worked here [23]. Researchers from the Institute of Botany (Tartu, Estonia), the Finnish Environment Institute (Helsinki), the University of Oulu, the University of Copenhagen, the University of Innsbruck, the University of Troms, the V.L. Komarov Botanical Institute (St. Petersburg), and many other Russian scientific organizations carried out their research work on the slope of the mountain. 

The Polar Urals in general, and Slantsevaya Mountain in particular, are located on the polar border of woody vegetation distribution. In the middle of the 20th century, forest–tundra spruce–larch open forests developed on its slopes; the entire territory is located in the permafrost zone. Due to that reason, a small number of fungal species were identified here [15]. Over 60 years, due to climate warming, the vegetation has transformed into the northern boreal. In the valley of the Sob River and along the mountain foot, the species of fungi previously found 250–350 km to the south have spread. For example, over half a century, the number of “aphyllophoroid” species has doubled [15]. Therefore, we take into account in our study the species that were collected on the mountain slopes from the 1960s to 2020. Thus, species that have appeared on the mountain over the past 20–50 years were excluded from this study. We also excluded species that were known in the 1960s but represented by single specimens, which did not allow us to estimate their range size on the mountainside. All species of mushrooms in this work are well-known to specialists and amateurs at the present time and 60 years ago. These mushrooms are characterized, predominantly, by large and prominent fruiting bodies. The definition of fungi was carried out using the following keys (Table S2).

We do not have any reliable data for every year between 1960 and 2020. We have the most complete data for 23 years, evenly distributed over the studied 60-year range. 

The data we have accumulated consist of collections of various scientific organizations, personal data of professional mycologists, and amateur but verified collections. The richest data were found for 66 species of Agaricomycetous macromycetes, and each of these species was found more than 100 times. All species were subdivided into three trophic groups (mycorrhiza formers, saprobes on litter and soil, and saprobes on wood), each comprising 22 species (Table S2). The collection altitude (m a.s.l.) is set for each specimen, the average altitude and dynamics for 60 years are set for each species. Fungal names are consistent with the IndexFungorum database [24]. 

### 2.3. Statistical Analysis

Linear mixed-effect models (LMM) were used to quantify changes in fruiting heights of individual mushroom species and three trophic groups over time. The following variants of the models were used: LMM with a random effect for individual species and trophic groups, as well as LMM with species-specific temporal trends and random intercepts on a species level. The response variable in each model was the average fungal fruiting altitude for each species in each year, and the predictive variable was the fruiting year. All models have been completed with a Bayesian framework using the brms package in R [25].

## 3. Results

**Figure 2:**
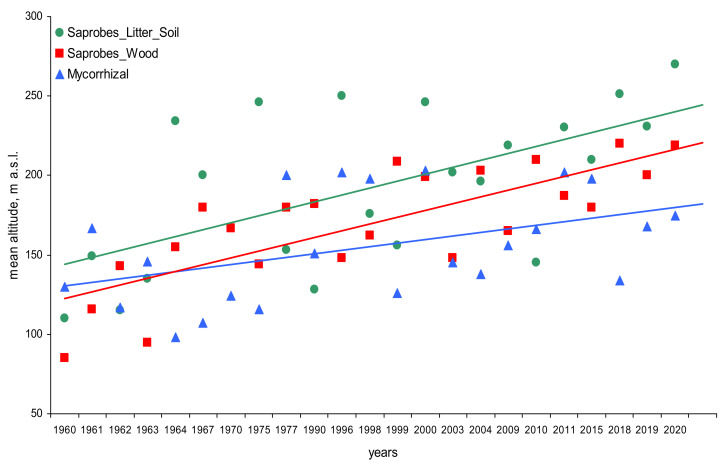
Trends in the mean altitude of fungal fruiting over time for saprobes on litter and soil (green circles; r = 0.51, *p* = 0.49), saprobes on wood (red squares; r = 0.50, *p* = 0.47), and ectomycorrhizal species (blue triangles; r = 0.23, *p* = 0.27).

**Figure 3:**
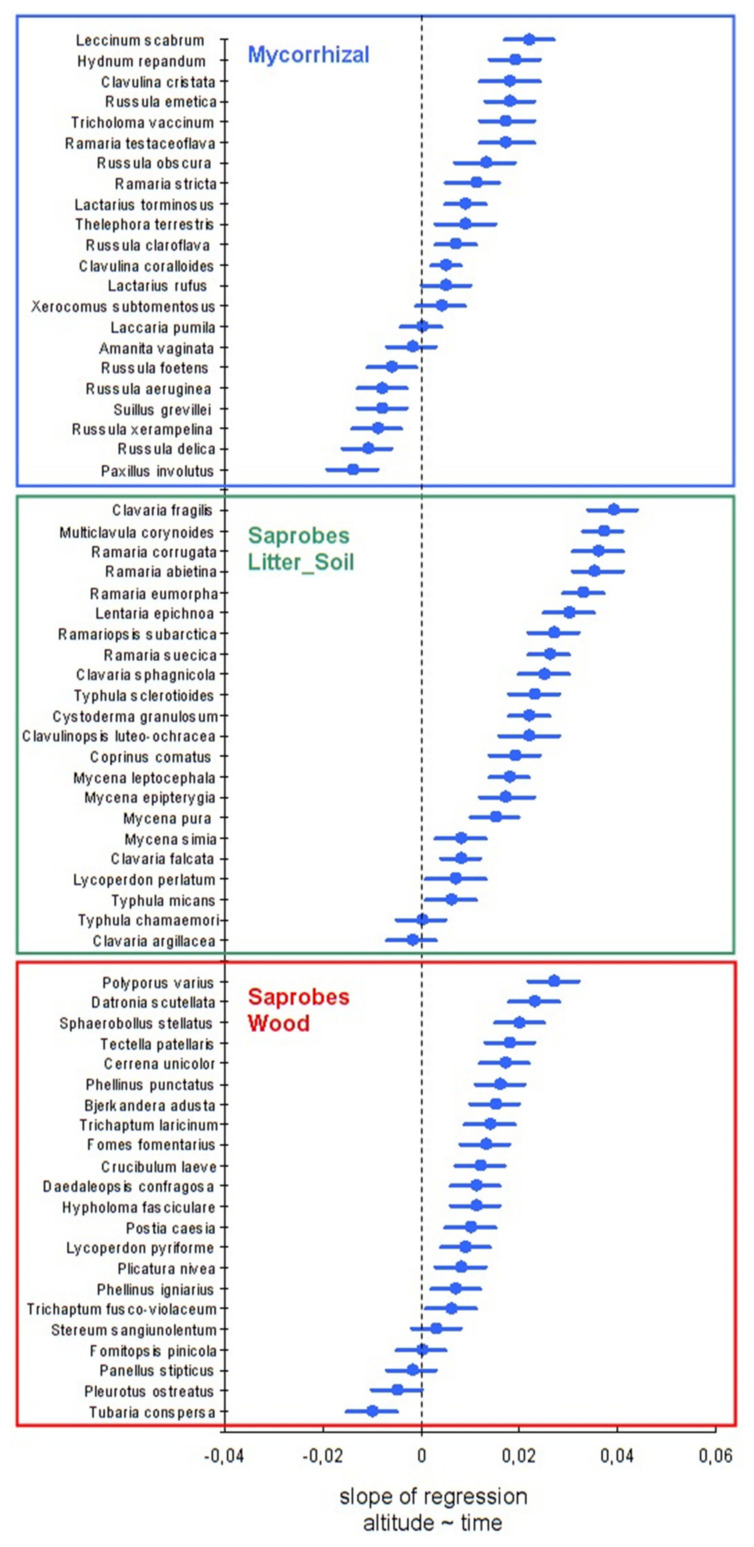
Species-specific trends over time at medium fruiting altitudes, where species are grouped according to their trophic groups. Mean and 95% confidence intervals are shown; the species to the right of the zero line tended to be collected at higher elevations during the 1960–2020 period.

All three trophic groups have increased the fruiting altitude over time (Figure 2). The linear trend of dynamics over 60 years for three trophic groups indicates that saprobes of litter and ground ascend most actively, on average by 1.6 m/year, of saprobes on wood at a rate of 1.5 m/year, while mycorrhiza formers are two times slower (0.8 m/year). In general, over 60 years, the average fruiting range of litter saprobes species has risen from 144 m above sea level up to 240 m a.s.l., saprobes on wood from 122 to 215 m, and mycorrhiza formers from 130 to 178 m a.s.l. Many individual species of fungi from these different trophic groups also showed significant tendencies towards an increase in altitude of fruiting above sea level over 60 years (Figure 3). Consequently, many species and groups of fungi have signs of increased altitudinal distribution, which may be associated with climate warming in the Polar Urals. Significant trends were observed over time for all groups.

Free-living saprobes on litter and soil showed the most obvious shift in fruiting up the mountain (Figure 3, Figure S3). In contrast, ectomycorrhizal fungi, closely associated with trees and thus limited by the distribution of host plants, showed the slowest shift. At the same time, saprobes on wood demonstrate an intermediate response rate in relation to the two previous groups. 

Of the 22 saprobe species on the litter and soil, over time, 20 species have increased in altitude (14 of them are significant; *p* < 0.05). Saprobes on wood react similarly, having perennial basidiomas, in which 18 species also increased in altitude over time (14 are statistically significant). However, out of 22 mycorrhizal species, 16 reacted positively to warming (nine species are statistically significant).

**Figure 4:**
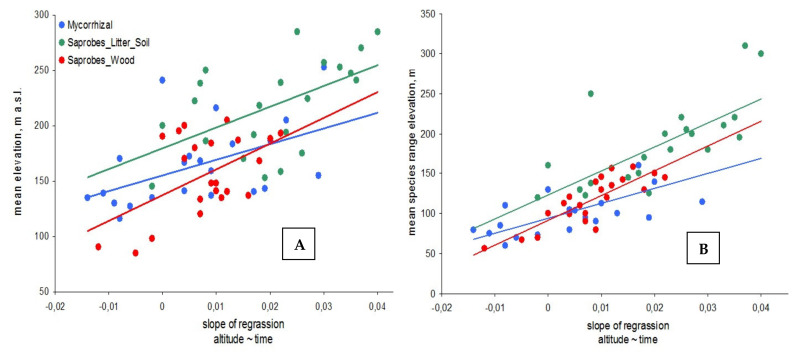
Species relationships between the altitude trend over time and mean fruiting elevation (**A**) of 1960 to 2020, and their altitudinal range (**B**). This shows how much the species move up or down on mean elevation (moving up if the slope is greater than 0, and down if the slope is <0). (**A**): saprobes on litter and soil (green circles; r = 0.54, *p* = 0.47), saprobes on wood (red; r = 0.47, *p* = 0.44) and ectomycorrhizal species (blue; r = 0.25, *p* = 0.29; (**B**): saprobes on litter and soil (green circles; r = 0.60, *p* = 0.56), saprobes on wood (red; r = 0.52, *p* = 0.61) and ectomycorrhizal species (blue; r = 0.22, *p* = 0.27).

Fungal species with a higher altitudinal distribution show stronger upward shifts (Figure 4A). Likewise, species with wider altitude ranges of fruiting also had a greater upward shift (Figure 4B). Again, we observed similar trends for mycorrhizal and saprobic fungi for these relationships. The alpine species may react more strongly to changes in the environment. Species fruiting at high altitudes may also be better adapted to temperature stress and frost than species fruiting at low altitudes in more stable conditions. Probably, due to their higher stress resistance, alpine species may be more susceptible to environmental changes. 

A similar trend was observed for plants and insects [12,18,19,20,26], where thermophiles species did not climb higher than expected, while alpine species became more common on the summits. In support of the generality of this trend, it was observed that plant and insect species with alpine distribution shifted more sharply in altitudinal distribution compared to species with more ubiquitous distribution [20,26], and lowland species experience a greater “lag” in response due to higher climate change rate in steeper topography conditions [27]. However, a stronger shift in the fruiting patterns of high-altitude fungi may also be simply a consequence of anomalous warming in the alpine belt of the Polar Urals [28,29] rather than due to differences in the ecology of individual species.

**Figure 5:**
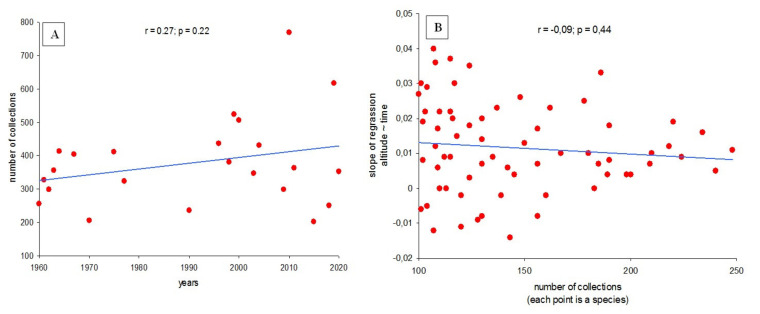
Total collections over 23 years (**A**) and slope of species’ estimated trends over time, as a function of their collection effort (**B**).

However, it is known that a range shift is possible when estimating based on historical observations, provided that the number of records for each species increases significantly over time [30]. Our dataset lacks a statistically significant increase in the number of records for each of the 66 species over the period 1960–2020 (Figure 5A). This indicates that sampling intensity as such does not appear to affect the estimated trends. In addition, if changes in the sample dynamics with respect to altitude were a strong factor in the observed patterns, then a fairly homogeneous response could be expected among different species, which was not the case. Although most species show an increase in average altitude of fruiting, some species nevertheless show the opposite trend. The observed variations in species-specific responses are in good agreement with those observed previously in studies of fungal phenology [4,5] and emphasize that analysis on the community level (Figure 2) can mask highly variable species-specific responses (Figure 3). Additionally, the proportion of collections exceeding the average volume did not increase over time, indicating a lack of a systematic propensity for higher collections over time (Figure 5B).

## 4. Discussion

The slopes of Slantsevaya Mountain demonstrate that over the past 60 years, the rate of microbiological decomposition of the litter has increased by 2–3 times [16], which is largely due to the appearance of new species of litter saprobes, which were previously found only at the base of the mountain. At the same time, in the middle and upper parts of the slope, the depth of seasonal thawing of permafrost has increased by 1–1.5 m [15], which probably contributes to the upslope movement of mycorrhizal species. However, it is possible that even such high growth rates of the seasonal permafrost drift turn out to be less meaningful compared to the sharp increase in the aboveground wood biomass and the rise of the upper forest boundary to the mountains [15], which encourages the spreading of saprobes on wood. Since 1960, the upper forest boundary on Slantsevaya Mountain has risen by 50–60 m, the forested area has grown by a third, the biomass of spruce has grown 21 times, and the biomass of larch 3.5 times, and therefore, the crown density has increased by 15–20%, which led to a 10–20% increase of the NDVI [15] (Table S1). Large deadwood of spruce and larch is now found much higher than in the 1960s, which preconditions the development of many species of saprobes on wood with large basidiomas at altitudes where they were undoubtedly absent before. Many wood saprobes are host-dependent or associated with a certain range of tree species, which may to some extent explain their more limited upward responses [15,16].

Mycorrhiza formers may also have failed to climb high up the slope due to the fact that new tree species never appeared, while larch, spruce, birch, alder, and mountain ash grew along the entire length of the transect 60 years ago already. Since 1960, their biomass and crown density have only increased. In this regard, the species richness of mycorrhizal fungi at the treeline is still low, although the number of some of them has increased.

It is also possible that some of the “positive” bioclimatic parameters listed above prevent a lesser rise in the mountains of mycorrhizal species. For example, the amount of precipitation on the mountain increases in winter and summer, the average annual temperature grows, and the permafrost thaws even deeper. In other words, because of warming, the slope becomes more humid due to permafrost melting and increasing precipitation. Where there were relatively dry slopes 30–40 years ago, streams are now constantly running, young bogs have formed on relatively leveled areas, and a pronounced swamping occurs at the base of the mountain. At many points at the base of the mountain, there is a massive decay of the root system of trees; they die off, and the meadow–forest vegetation changes to lakeside–bog. The area of Sphagnum bogs has increased dramatically.

At the same time, in the middle part of the slope, the maximum number of macromycete species has been identified [16], which we associate with “ideal” conditions: there is no stagnation of cold valley mountain air, which made it possible to identify here the maximum average daily temperatures in July–September; drainage conditions are the best here; and the number of species of woody and herbaceous plants is the richest. For saprobes on wood, these are excellent conditions, which is associated with the maximum level of aboveground biomass of woody ones, although 40–60 years ago, there were no large trees, let along any large deadwood, in the middle part of the slope [16] (Figure S1; Table S1). 

Comparison of our findings with conceptually close studies, for example, in the Alps [5], indicates that 1) in the Polar Urals and in the Alps, the fruiting of macromycetes shifts upward over time; 2) the shifts are specific for individual species and trophic groups: in both regions, the greatest shift has been found for saprobes on the litter and soil; however, in the Polar Urals, wood-dwelling saprobes rate second, and mycorrhizal organisms have demonstrated the least shift; and 3) in both regions, fungi that live and bear fruit at high altitudes are likely to be closer to their fundamental physiological limit, and such species respond stronger than species with a lower distribution.

Macromycetes with a high slope distribution are often characterized by an Arcto-Alpine range in Northern Eurasia. Such species have been shown to indicate warming in the Arctic, and they are regularly included in the corresponding Red Data Books [19]. On Slantsevaya Mountain, this group is among the leaders in raising the fruiting altitude (Figure 3). The species of this group have almost completely disappeared in the valley and at the slope foot and have also shifted upwards from the middle part of the slope—to the treeline and higher. This applies to the following species represented: *Clavaria sphagnicola* Boud., *Clavulinopsis luteo-ochracea* (Cavara) Corner, *Datronia scutellata* (Schwein.) Gilb. & Ryvarden, *Multiclavula vernalis* (Schwein.) R.H. Petersen, *Ramariopsis subarctica* Pilát, and *Typhula chamaemori* L. Holm & K. Holm. A similar trend was found for some other cryophilic species (*Multiclavula corynoides* (Peck) R.H. Petersen, *Peniophora aurantiaca* (Bres.) Höhn. & Litsch., *Sclerotrema griseobrunneum* (K. Wells & Raitv.) Spirin & Malysheva, *Typhula tremula* (Berther) Olariaga, *T. ishikariensis* S. Imai, *T. incarnata* Lasch). Moreover, the last two species are plant pathogens that cause the “snow mold” disease of cereals [31]. Consequently, fungal plant pathogens are already waiting for the expansion of the crop production zone to the north.

It should be noted that over the past 20 years, subarctic species, known only in Asian natural habitats (*Cerioporus choseniae* (Vassilkov) Zmitr. & Kovalenko, *Clavariadelphus mucronatus* V.L. Wells & Kempton), have been introduced into the middle and lower parts of the mountain [15]. On the other hand, the bioclimatic conditions of the region became so comfortable that in 2018, basidiomas of *Polyporus umbellatus* (Pers.) Fr. were collected at the foot of Slantsevaya Mountain. This one is associated with oak-beech forests in Europe and boreal spruce–larch forests in the Siberian taiga [16].

East of the Polar Urals, the Yamal Peninsula is located, where the abundance dynamics of Agaricomycetous saprobes on wood was studied [32]. Over 40 years, the average annual air temperature in the study area has increased by 0.8 °C, which caused the “forest–tundra” population of model fungi species to shift to the north. For Agaricomycetous macromycetes growing on deciduous wood in zonal habitats, the displacement of fruiting averaged 47 km/1 °C, while those associated with evergreen coniferous averaged 31.5 km/1 °C.

## 5. Conclusions

Monitoring mycological studies on the altitudinal transect “Slantsevaya Mountain” have been underway for 60 years. Here, a tendency to an increase in the fruiting altitude of the three trophic groups of Agaricomycetous macrofungi was revealed. The results are confirmed by annual data. Similar results for individual species and trophic groups have been established for other mountainous regions of Europe. Nevertheless, some differences were also revealed: in the Polar Urals, mycorrhizal fungi showed the least uplift, while, for example, in the Alps, saprobes on wood showed the least uplift. In both regions, the maximum elevation was established for saprobes on litter and soil.

It should be noted that the collections of fungal fruit bodies only indicate the presence of a species, i.e., represent only a small but important part of the life cycle of fungi associated with sexual reproduction. We expect that the patterns identified using large datasets on the number of harvested fruiting bodies reflect parallel changes in belowground activity and distribution as these fungi complete their life cycle and multiply by spores that spread from the fruit bodies. Currently, DNA-based analysis is an important tool in the study of fungal ecology for understanding the structure and functioning of the belowground part of the fungal community. However, since soil samples in the Polar Urals were not collected 40–60 years ago, observation of basidiomas is currently the only way to assess the long-term altitudinal dynamics of changes in fungal fruiting.

This study shows that macromycetes are indicators of climate changes in the Arctic. At the same time, the Arcto-Alpine species are among the clearest indicators, which retreat even higher into the mountains as the climate warms up and have either completely disappeared or sharply reduced their population at the foot of the mountains.

## Data Availability

The climatic data and fungal species dynamics (1960–2020) discussed in this study are available in Shiryaev et al., 2019, 2020, as well as the fungal species list presented in Supplementary Materials.

## Supplementary Materials

The following are available online at https://www.mdpi.com/article/10.3390/microorganisms9091892/s1, Figure S1. Upshift of treeline and crown density 1962–2020 on the Southern slope of Slantsevaya mountain; Figure S2. (A) Upshift of the timberline from 1962 to 2018 years (investigated transect marker as a gray rectangle) and (B) NDVI grow from 1982 to 2018 years; Figure S3. Mean changes of three trophic groups (ectomycorrhizal, saprobes on litter and soil, saprobes on wood) on the Slansevaya Mountain; Table S1. Changes of biolimatic parameters on the Southern slope of Slansevaya mountain; Table S2. Table of fungal species information.
